# Effects of exploring a novel environment on memory across the lifespan

**DOI:** 10.1038/s41598-022-20562-4

**Published:** 2022-10-05

**Authors:** Judith Schomaker, Valentin Baumann, Marit F. L. Ruitenberg

**Affiliations:** 1 Department Health, Medical and Neuropsychology, Leiden University, Leiden, The Netherlands; 2 Leiden Institute for Brain and Cognition, Leiden, The Netherlands; 3 Department of Child and Adolescent Psychiatry and Psychotherapy, University of Magdeburg, Magdeburg, Germany

**Keywords:** Cognitive ageing, Cognitive neuroscience, Learning and memory, Motivation

## Abstract

Exploration of a novel environment has been shown to promote memory formation in healthy adults. Studies in animals have suggested that such novelty-induced memory boosts are mediated by hippocampal dopamine. The dopaminergic system is known to develop and deteriorate over the lifespan, but so far, the effects of novelty on memory across the lifespan have not yet been investigated. In the current study, we had children, adolescents, younger, and older adults (*n* = 439) explore novel and previously familiarized virtual environments to pinpoint the effects of spatial novelty on declarative memory in humans across different age groups. After exploration, words were presented while participants performed a deep or shallow encoding task. Incidental memory was quantified in a surprise test. Results showed that participants in the deep encoding condition remembered more words than those in the shallow condition, while novelty did not influence this effect. Interestingly, however, children, adolescents and younger adults benefitted from exploring a novel compared to a familiar environment as evidenced by better word recall, while these effects were absent in older adults. Our findings suggest that the beneficial effects of novelty on memory follow the deterioration of neural pathways involved in novelty-related processes across the lifespan.

## Introduction

Do you remember what you had for breakfast during your holiday abroad last year? And do you remember what you had for breakfast the day after getting back? Chances are that you have recollection of your holiday breakfast, but not of the one you had at home. Although both events are almost equally distant in time, the breakfast you had in a familiar environment was less memorable than the one you had in an unfamiliar holiday location. From an evolutionary perspective it makes sense that learning is promoted around the time that novel environments are explored. When visiting a new place, it is crucial to learn about the circumstances of danger and reward, to optimize chances of avoiding danger and finding rewards in the future^1^. Also events occurring in the temporal vicinity of novelty exploration are relevant, as these may relate to the requirements or consequences of exploring that environment.

Studies in rodents have robustly shown that novel environments not only promote learning in these environments but can also promote long-term potentiation—a neural mechanism linked to memory, for events occurring before or after novelty exposure^2–4^. Animal work has further suggested that the effects are dependent on hippocampal dopamine that can have two potential sources: One being the ventral tegmental area (VTA) and the other being the locus coeruleus (LC;^5–8^. Novelty has been suggested to be a behaviorally relevant event, that can especially push weak memory traces towards memory persistence^9–13^. For example, weak but not strong taste memory has been shown to be facilitated by novelty^14^, and novelty has been suggested to boost memory persistence for weak memory that would else not be consolidated^5^.

Relative to the work in animals, however, research on the effects of novelty on memory is lagging in humans^15^. However, a handful of studies have suggested that similar effects exist in humans too. In the first study that investigated the effects of novelty on memory in humans, adult participants were exposed to pictures of novel or familiar (i.e., repeated) scenes before performing a word learning task 5 min later^10^. Commensurate with the findings in animals that suggest that novelty can promote memory, it was observed that participants recollected more words on this unrelated task, 24 h later after being exposed to novel rather than familiar stimuli. This study observed effects of novelty on consolidation, but also on encoding. Previous neuroimaging work has suggested that co-activation of the hippocampus and dopaminergic midbrain support successful encoding (e.g.,^16–18^, and it has been suggested that phasic dopamine during initial learning can enhance memory independent from consolidation^19^. As novelty was found to activate dopamine-related regions (including the substantia nigra/ventral tegmental area) it is possible that novelty may mediate memory through effects on encoding or consolidation^10^. The study by Fenker et al.^10^, however, contrasts with the previous work in animals that investigated effects of novelty on memory, in that it employed images of novel scenes rather than novel environments. More akin to the studies in rodents, one study used 3D immersive novel and familiar environments by employing virtual reality^20^. In a within-subjects design, participants explored a novel environment on one day and a previously familiarized environment on a different day, before performing a word learning task. It was found that exploration of a novel environment improved recall, but not recognition in a memory test shortly later.

Not only novel scenes and spatial novelty have been shown to have benefits on memory. One study in elementary school children found that experiencing a novel but not familiar science or music lesson 1 h before or after reading a story improved memory of the story^11^. Similar beneficial effects of novelty have been found on visual memory in adolescents, suggesting that experiencing novelty can have a generalizable effect on long-term memory^21^. In line with the work in healthy adults^20^, one previous study employed spatial novelty, comparing effects in children and adolescents with attention deficit hyperactivity disorder (ADHD) and a typically developing control group^22^. Findings from this study suggest that exploration of a virtual novel environment improved free recall on a word learning task in children and adolescents with ADHD, but not in typically developing children. The authors suggested that the lack of positive effects in the control group may be explained by novelty only improving weak memory traces, which may have been more prevalent in the children with ADHD due to general learning impairments, but the role of memory strength has not yet been experimentally investigated in humans.

Notwithstanding these prior indications of differences between children and adults, the effects of spatial novelty on memory across the lifespan has not been investigated systematically, and it thus remains unclear whether age influences these effects. Psychophysiological studies have suggested that the topology and size of the novelty-induced event-related components change with aging^23–30^, and one previous study found that children respond stronger to stimulus novelty than adults, as evidenced by faster responses to an auditory target when a novel rather than familiar image was shown^31^. Changes in the response to novelty across the lifespan are supported by structural and neuroimaging studies that have suggested an age-related degeneration of the substantia nigra/ventral tegmental area (SN/VTA;^32^ and locus coeruleus^33,34^, which are the two potential sources of hippocampal dopamine believed to underlie the effects of novelty on memory consolidation^5–8^. The age-related degeneration of these regions could also potentially reduce the beneficial effects of novelty on memory. So far, only a limited number of studies have investigated the effects of novelty on memory over the lifespan. One study that investigated effects of novelty in a healthily aging population did not observe positive effects of novelty^35^. In this study, the novel condition included *passive* exposure to novel nature videos, and the authors argued that active interaction with the novel material may be required (also see^36^). In line with this suggestion, a recent study in young adults suggested that *active* exploration of novel environments may indeed be required, as only active exploration, but not passive novelty exposure enhanced word recall^37^. Novelty-exposure interventions have been argued to have the potential to counteract or slow-down age-related memory decline^15,32^, but as the influence of age remains underinvestigated it is currently unclear whether the beneficial effects of novelty on memory as discussed above are also present in older adults.

In the current study we tested the effects of novelty on memory over the lifespan, in a relatively large sample (*n* = 439) including children (8–11 years), adolescents (12–17 years), younger adults (18–44 years), and older adults (45 years or older; age groups divided to fit the dopaminergic functioning curve across the lifespan^38^. We furthermore aimed to investigate whether the effects of novelty depend on the strength of the memory trace. For this purpose, participants first were familiarized with one of two virtual environments (VEs) that they could freely explore. They then explored the same (familiar) or a novel environment, after which they were shown a list of words. One group of participants performed a *deep* encoding task (“does the shown word reflect something that is alive or not?”), while another group of participants performed a *shallow* encoding task (“is the first letter of the shown word open or closed?”). After a short distraction task, word recall and recognition, and memory for encountered landmarks during exploration was tested. Participants also filled out a novelty seeking (NS) questionnaire^39,40^.

We expected that novelty exploration would promote word recall but not recognition (cf. ^20,37,41–43^). We furthermore expected that these effects would be stronger in adolescents and younger adults as compared to children and older adults, since dopaminergic functioning rises and declines with age^22,38^. As novelty has been shown to especially promote encoding of weak memory traces in animals^5,14,44^, we explored whether the boosting of novelty would be stronger for participants who performed the shallow rather than deep encoding task^5^. We furthermore expected that exploration behavior would be positively linked to subsequent word recall, via putative dopaminergic modulation^32^.

## Materials and methods

### Participants

A total of 487 visitors of the NEMO Science Center in Amsterdam aged 8 years or older volunteered to participate in this study. Data was collected during a 2-week Science Live exhibition, during which we tested all visitors interested in volunteering during all opening hours of the NEMO Science Center. While this somewhat restricted our control over the age and the total number of participants, it yielded a final sample size that largely exceeded that of prior studies (e.g., between 30 and 103 participants in references^25^ and^28^). Forty-five participants were excluded: 17 participants were excluded because of administrative issues (e.g., accidental reuse of a participant number), seven due to technical issues (e.g., task crash), seven because of language issues (e.g., unable to understand the instructions), six because they worked together or received help from a parent, five participants because they did not finish the tasks in sequence (e.g., with a long break to visit an exhibition show), and two participants because they talked on the phone during the word learning task. As such, 439 participants were included in the main analyses (401 performed the task in Dutch and 38 in English). As the landmark test did not run on all laptops due to technical issues, the number of included participants that completed this task was only 331. Participants were classified as children (8–11 years; mean = 9.33; SD = 1.15), adolescents (12–17 years; mean = 13.19; SD = 1.43), younger adults (18–44 years; mean = 32.73; SD = 8.27) or older adults (> = 45 years [range 46–77]; mean = 53.30; SD = 8.23 ) based on their age (and presumed associated differences changes in dopaminergic functioning:^38,45,46^). Supplementary Information (SI): Appendix 1 shows demographics and the distribution of participants over age groups and conditions. Participants in the first testing week performed a word learning task with a deep encoding, and participants in the second week performed a shallow encoding task. For participants within each age-group, age distributions were similar across the different novelty and level of processing conditions (for novelty and level of processing respectively, children: *p* = 0.598 and *p* = 0.405; adolescents: *p* = 0.568 and *p* = 0.155; young adults: *p* = 0.077 and *p* = 0.815; old adults: *p* = 0.658 and *p* = 0.733). Also sex distributions were similar over conditions (Pearson Chi-Square for novelty and level of processing respectively, children: *p* = 0.216 and *p* = 0.821; adolescents: *p* = 1 and *p* = 0.128; young adults: *p* = 0.214 and *p* = 0.853; older adults: *p* = 0.285 and *p* = 0.241).

All participants or a participant’s parent in case of minors, gave written informed consent. Participants could choose to perform the tasks in Dutch or English. The study was approved by the Psychology Research Ethics committee (CEP) of Leiden University, the Netherlands. All procedures were in line with the Declaration of Helsinki (1964, and later amendments), and followed relevant COVID-19 guidelines and regulations.

### General procedure

Throughout all procedures the experimenters were wearing a mask and gloves as a safety regulation regarding the COVID-19 pandemic. For data collection we used six laptops in two spacious testing rooms that allowed for social distancing (> = 1.5 m). The experimenter stayed in the testing room throughout the entire procedure to start the tasks and to answer questions. The entire experimental procedure took approximately 15–25 min.

Data was collected at the NEMO Science Center in Amsterdam. Upon arrival, participants were asked to disinfect their hands as part of the COVID-19 protocol. Before participation, participants or their parents read the information letter and were given the opportunity to ask questions. After giving written informed consent, the participants were seated before they performed a series of tasks on a laptop.

### Stimuli and apparatus

**Figure 1:**
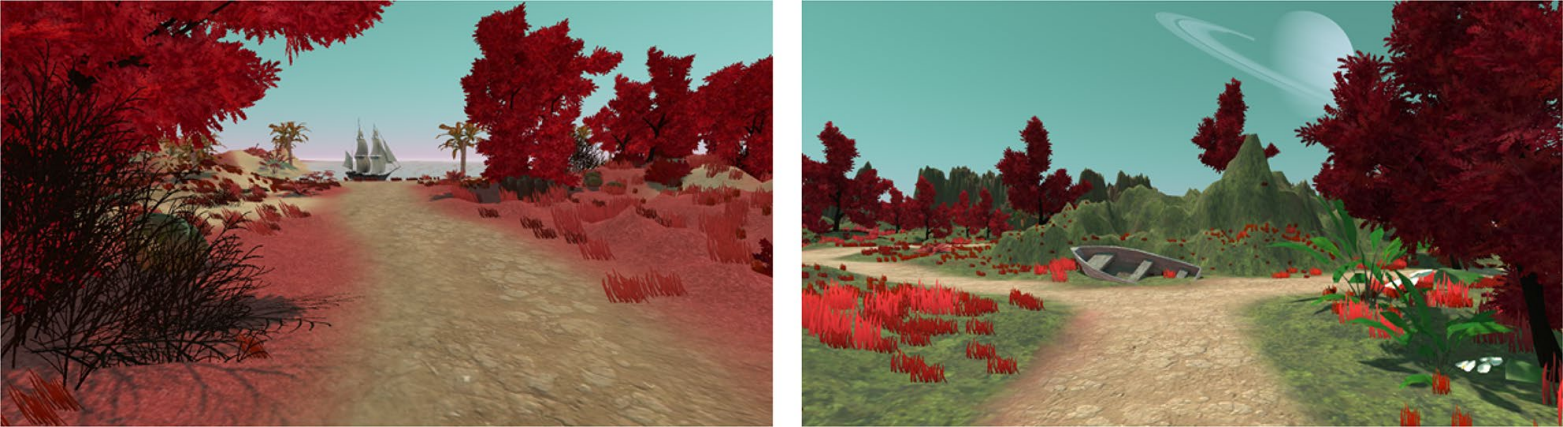
Screenshots of the two virtual environments. The environments contained landmarks at intersections and road endpoints, and were matched in size, number of intersections, number of landmarks, and path length.

The VEs were created using Unity Version 2017.2.21f1 (Unity Technologies, 2017), and were matched in size, path length, and number of intersections. Both VEs consisted of fantasy islands with unusual landmarks (such as a slot machine) at intersections or road endpoints, including land and a body of water (see Fig. 1). The VEs were presented on laptops running on Windows 10 (Microsoft, 2015). Participants could move forward using the W key on the keyboard and the mouse to determine the heading direction. During exploration the X, Y and Z coordinates of the moving agent were logged for all timepoints with a sampling rate of about 15 Hz. The VAS I, VAS 2 and word learning task were programmed and presented using Open Sesame 3.3.3^47^, the landmark task and NS questionnaire were created using E-Prime 3.0 software (Psychology Software Tools, Pittsburgh, PA).

For the word learning task fifteen Dutch neutral nouns were chosen from the CELEX lexical database and translated to English for non-Dutch speakers^48^. The same words were used in the novel and familiar, and shallow and deep encoding conditions. Four words referred to an animal (“alive”) and eleven words referred to a non-living thing (“not alive”). Similarly, four words started with a closed letter (e.g., “Boat”) and eleven with an open letter (e.g., “Wolf”).

Participants were reminded of the response keys and task during the encoding, recall and recognition phase of the word learning and landmark tasks. The response keys were shown below the word, in the location corresponding to the keyboard, and in the semantic task the response keys were further accompanied by the picture of a cow (to indicate a living thing) and a chair (to indicate a non-living thing). These reminders were included to lift the working memory load, especially because this otherwise could have made the task disproportionally difficult for the younger children.

Landmarks were objects from the Unity Asset store, and included a wide range of easily recognizable objects, such as an airplane and desk chair. Pictures of the landmarks presented on a grey background were used in the landmark memory test. During this test also lures were presented, which consisted of objects that were not part of either of the two VEs.

### Exploration phases and affective ratings

Participants received scripted verbal instructions regarding how to navigate through the VE. The ‘W’ key (for ‘walk’) could be used to move forward, and the mouse could be used to look around and determine heading direction. The space bar could be used to jump, although there was no function in jumping, as one could not jump on top of things. Participants were instructed that they could navigate freely but should try to stay on the paths. During the first *familiarization phase*, participants explored the VE for 3 min. After exploration, they were asked to indicate their happiness (“How happy are you?”, from 1 = extremely unhappy to 9 = extremely happy) and arousal (“How aroused are you?”, from 1 = very calm to 9 = very excited) on a *visual analogue scale* (VAS) with Self-Assessment Manikins^49^. They could use the number keys to indicate their answers, and completing the ratings took less than 1 min.

**Figure 2:**
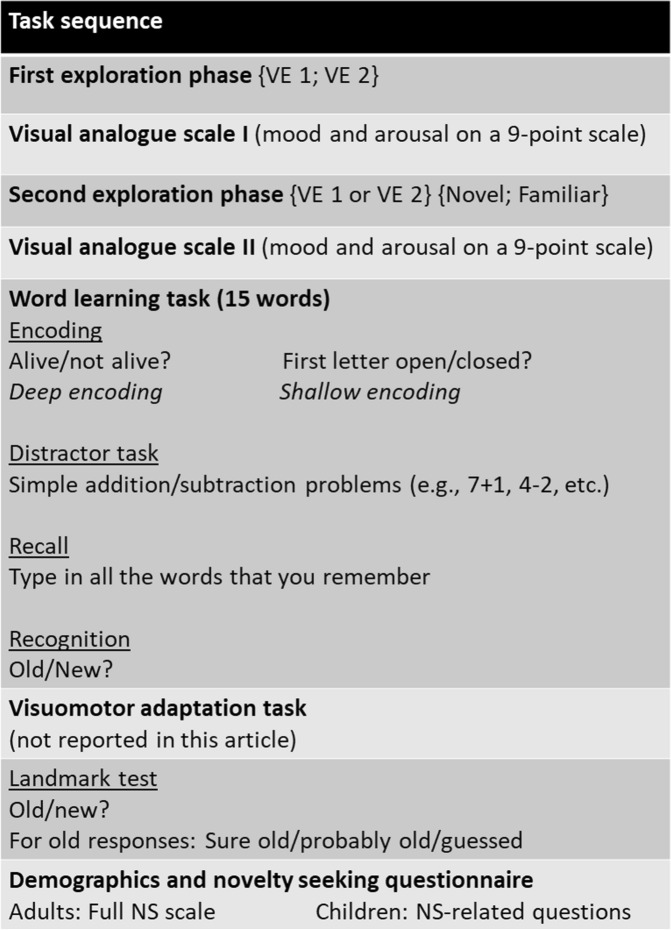
Experimental task sequence. Tasks are shown in sequential order from top to bottom. During the first exploration phase participants explored one of the two virtual environments (counterbalanced between participants). Participants filled out Visual Analogue Scales to report current mood and arousal state^44^ In the second exploration phase participants either explored the same (familiar condition) environment again or a new one (novel condition). The depth of encoding during the word task was varied between subjects, with participants either performing a semantic (deep encoding condition) or shallow encoding task. After a short distractor task, memory ways tested with free recall and a recognition test. After a visuomotor adaptation task (not reported here) landmark memory was tested with a recognition test with confidence judgments. Finally, adults filled out the full Novelty Seeking scale of the TPQ^35,36,45^, while children answered NS-related questions (non-standardized).

During the *second exploration phase* participants explored either the same (i.e., familiar) or a new VE for another 3 min (i.e., novelty and VEs were counterbalanced). After this exploration, participants were asked to rate their happiness and arousal levels again on the same two *VAS* as before the first exploration. See Fig. 2 for the experimental task sequence.

### Experimental tasks

For the *word task*, instructions were shown on the screen. During the encoding phase, fifteen nouns were shown in a random sequence (we believe this number of items to be sufficient to identify individual and condition differences, as the smaller 10-word learning list from the Consortium to Establish a Registry for Alzheimer’s Disease [CERAD] has been shown to be a sensitive measure for detecting mild cognitive impairment and identifying early symptoms of Alzheimer’s disease, suggesting that relatively short word lists are sufficient to robustly identify individual differences in memory performance^50,51^. Also other neuropsychological test batteries use relatively short word lists, such as the California Verbal Learning Test [CVLT;^52^] which uses 16 words, or the Rey Auditory Verbal Learning Test [R-AVLT] which uses 15 words^53^). In the first week of data collection, word learning involved a *deep* encoding task in which participants had to judge whether the shown word represented a living (e.g., a cow) or a non-living (e.g., a chair) thing. During the second test week, word learning involved a *shallow* encoding task in which participants had to indicate whether the first letter of the shown word had an open (such as a “W”) or closed (such as an “O”) shape. Each word was presented for a duration of 3000 ms (irrespective of whether a response was given or not). In between words a fixation cross was shown for 500 ms. After the encoding phase, participants performed a series of nine simple math problems (e.g., 4 – 3 or 7 + 1) in a *distractor task*. The solution to all problems varied between 1 and 9. Next, participants were prompted to enter as many words as they could remember from the encoding phase. They were instructed to press ENTER, to continue entering words or to press ESC + ENTER to continue if they could not remember any more words. In the following *recognition test* all 15 words from the encoding phase were randomly shown, interspersed with 10 lures (new words that were not presented during encoding). Participants had to indicate for each word whether it was old (“press X”) or new (“press N”). Each word was shown until a response was given. All phases of the word task were finished in 3–4 min. Recall was quantified by the percentage correctly remembered words, while recognition was quantified by the corrected hit rate (CHR = percentage old hits – percentage new false alarms). Next, participants performed a *visuomotor adaptation task*, which was completed in 2–3 min (results published in^54^).

The *landmark test* assessed memory for landmarks that participants could have encountered during the second exploration phase. In total 35 landmarks were shown, of which 20 were present in the second VE (i.e., “old) and 15 were lures (i.e., “new”). Participants had to indicate for each landmark whether they saw it before (“press X for old”) or not (“press N for new”). When participants indicated “old” they were further asked to indicate whether they thought the landmark was “sure old” (“press X”), “probably old” (“press N”) or whether they guessed (“press M “). Each landmark was shown until a response was given and the test had a duration of approximately 2–3 min. As an estimate of landmark recollection, the “sure” CHR was calculated (i.e., “sure” old hits—new false alarms).

### Novelty seeking questionnaire

Finally, participants reported their sex (male; female; other), age in years, and handedness (right; left; ambidextrous). Adults (> 17) subsequently filled out the 34 items of the *NS scale* of the Tridimensional Personality Questionnaire^39,40,55^, whereas children and adolescents filled out a simplified and abbreviated (20 item) version of the questionnaire. Each question remained on the screen until a response (“X” = yes; “M” = no) was given. All questions could be answered in about 2–5 min. Afterwards feedback was shown on basis of the total NS score (i.e., with a subdivision into low, medium, and high scorers). These cut-off scores were only used to provide the participants feedback and were not used in any analyses.

### Analyses

#### Memory performance

Recall and CHR for words were subjected to 2*2*4 ANOVAs with Novelty (novel; familiar), Encoding type (shallow; deep) and Age group (children; adolescents; younger adults; older adults) as between-subject factors. As we expected the effects of age on memory performance to be quadratic, with performance peaking in adolescents or young adults, we followed up a main effect of age group with a quadratic contrast^38^. In line with our hypothesis that older adults would show diminished effects of novelty, an interaction between novelty and age was followed up with three 2*2*2 ANOVAs with Novelty (novel; familiar), Encoding type (shallow; deep), and Age (either older adults vs. children, older adults vs. adolescents, or older vs. younger adults) as factors. As the groups between conditions were unequal, we also included Encoding type in this analysis, but the main effect and interactions with this factor are not interpreted. For all analyses, the α-criterion was set at 0.05, and Bonferroni-Holm correction was applied to compensate for multiple testing.

#### Roaming entropy, and other measures of exploration

**Figure 3:**
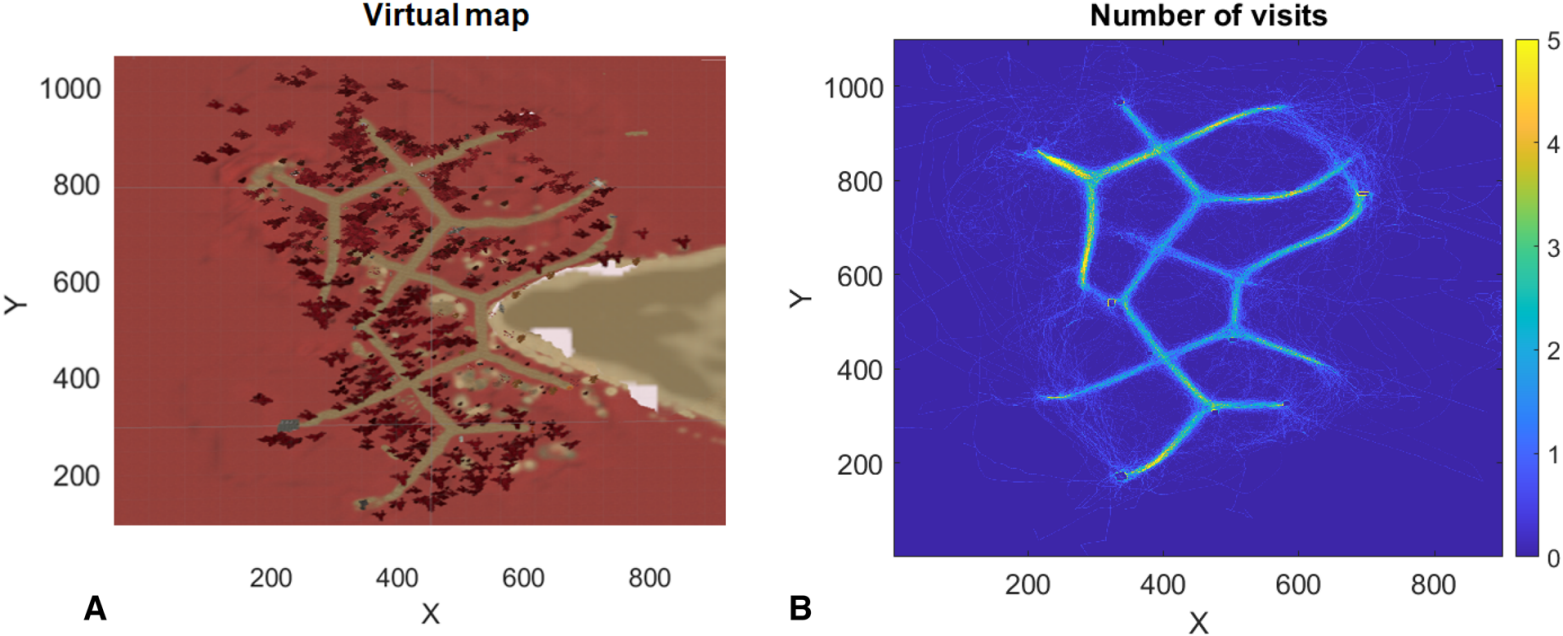
Maps of one of the virtual environments. (**A**) Depicts the map of one the VEs. (**B**) Shows the number of visits per XY-coordinate in a heatmap for all participants that explored that island. The spawn point in the top left is visible as a highly visited region. Outlines of landmarks can be recognized at some ends of paths. Individual navigation traces show that some people left the paths and used short-cuts to other paths. This data was used to calculate a probability matrix reflecting the likelihood that each of the locations was visited (see main text). Note, the number of visits per XY-coordinate is relatively low, despite smoothing. The probability that each location was visited was used to calculate the roaming entropy (RE) of individuals.

Roaming entropy (RE) during the first and second exploration round was defined for each participant. In this analysis the Z-coordinates were omitted, as the VEs consisted of only one location for each of the XY coordinates (although it was possible that people jumped at a location, they could not climb on anything). As there was a very high number (6.31 million) of possible locations the likelihood that the same coordinates were visited during the 3-min exploration was small, therefore the individual paths were smoothed using a Gaussian filter with a width of 100. Then a likelihood matrix *p*_*j*_ was calculated for each of the two VEs, where the likelihood that someone visited each of the XY positions *j* was calculated*,* by dividing the total number of visits to that location by the total number of visited locations for all participants. See Fig. 3A for the map of one of the VEs, and 3B for a heatmap depicting the number of visits for all XY coordinates for that VE.

Roaming entropy (*RE*_*i*_) was calculated per participant and exploration round by summating over the product between the individual’s path (*p*_*ij*_) and the log of the probability that each location was visited (*p*_*j*_) divided by the log of the number of possible locations (*k*):

$$RE_{i} = - \sum\limits_{j = 1}^{k} {\left( {\frac{{p_{ij} \log (p_{j} )}}{\log (k)}} \right)}$$

High RE indicates that the participant explored more of the less-often-walked paths, while a lower value reflects higher concordance to the often-walked paths.

In addition to RE, we calculated the total distance travelled (in Unity meters) as the sum of Euclidian distances between successive datapoints (2D) and counted the number of landmarks that were encountered in the second exploration round for each participant by defining regions of interest (ROIs) for each of the landmarks for which memory was tested. These ROIs consisted of rectangular bounding boxes around the landmarks. ROIs could overlap in case landmarks were close to each other. For each participant it was determined which ROIs were visited. The total number of ROIs visited provides an additional measure of exploration, as it reflects how many regions were visited by the participant. A GLM including novelty (novel; familiar) and encoding type (deep; shallow) as categorical predictors, and RE for round 2 and age as continuous predictors of word recall was ran, to investigate whether exploration behavior as quantified by RE could predict later word recall above and beyond the effects of novelty and encoding type. We chose to include only RE and not distance travelled, or landmarks encountered in this model, as these measures were found to be positively correlated (see SI: Appendix 5). RE is the most commonly used measure of exploration and the only of these three measures for which we found a novelty effect. The GLM was ran on centered data to reduce multicollinearity. Multicollinearity was shown to be low, with all variance inflation factor (VIF) values < 1.15. It is of note that we ran our task on different laptops with varying specifications, which resulted in different sampling rates between participants, but as participants were randomly distributed over the laptops, and RE exhibits a similar pattern of results as the other exploration measures (e.g., distance traveled, and landmarks encountered) we believe that potential effects of these differences were minimal.

### Preprint

A previous version of this manuscript was published as a preprint https://psyarxiv.com/r2tdn/

## Results

### Word recall

**Figure 4:**
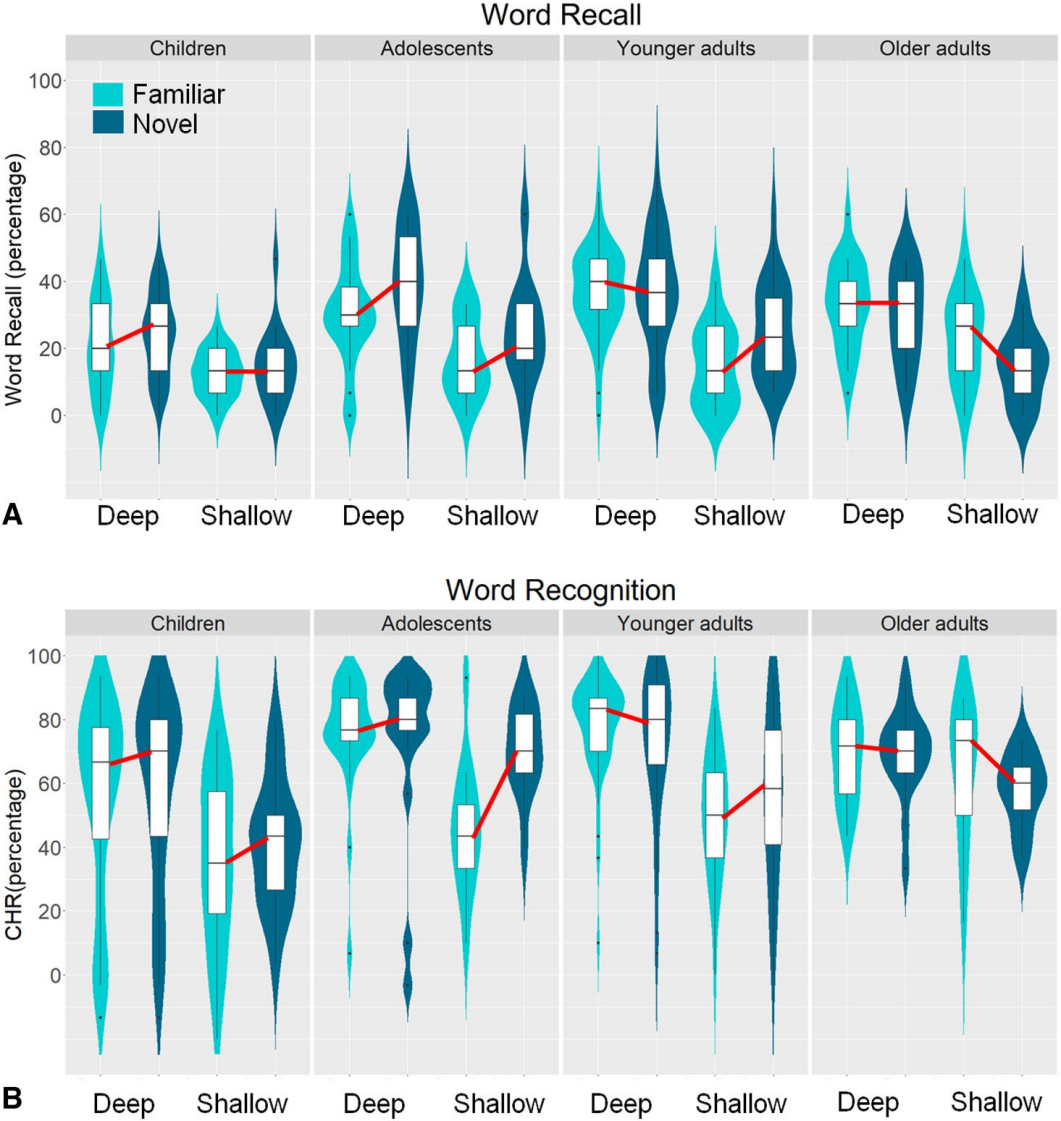
Memory performance on the word learning task. (**A**) Word recall and (**B**) word recognition as measured by CHR on the word learning task in percentages for children (age 8–11), adolescents (age 12–17), younger adults (18–45) and older adults (46–71) in the deep and shallow encoding conditions after exploring a novel or familiar environment. The red lines show the novelty detriment (line going down from left to right) or novelty bonus (line going up from left to right). There appear to be negative values for word recall, as the data was smoothed with a kernel density estimator. Note, however, that word recall ranged between 0 and 100%, while CHR could be negative when participants had more false alarms than hits.

SI: Appendix 2 reports analyses of subjective arousal and mood ratings. In SI: Appendix 3 the level-of-processing analyses for performance during encoding are reported. Figure 4A shows word recall per age group as a function of novelty condition and word encoding condition. The effects of novelty (novel; familiar), encoding type (shallow; deep) and age (children; adolescents; younger adults; older adults) on word recall and recognition were investigated with two univariate ANOVAs. Participants in the deep encoding condition recalled more words than participants in the shallow encoding condition, *F*(1, 413) = 76.72, *p* < 0.001, *ŋ*^2^ = 0.16. Word recall also differed as a function of age group, *F*(3, 413) = 14.33, *p* < 0.001, *ŋ*^2^ = 0.09. A quadratic relationship of age was observed, *Contrast estimate* = − 0.65, *p* < 0.001, with the adolescents and younger adults remembering more words than the children and older adults.

**Figure 5:**
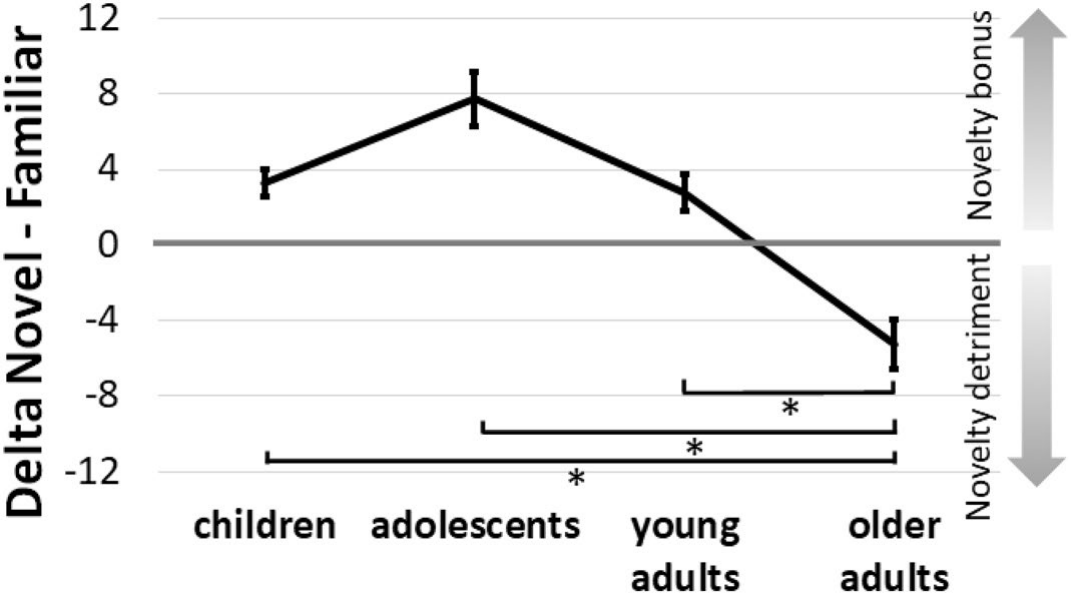
Effects of novelty on word recall for the different age groups. Mean word recall (%) for the novel minus familiar condition is shown for all age groups (children; adolescents, younger adults; older adults). Positive values reflect higher word recall after exploring a novel versus familiar environments (i.e., a novelty bonus), while negative values reflect a detriment after exploring a novel environment. Children, adolescents, and younger adults had higher word recall after exploring a novel, while older adults had higher word recall after exploring a familiar environment. Error bars reflect standard errors of the mean. Highlighted effects (*) reflect a significant interaction between novelty and the connected age groups (note, only comparisons between the older adults and the other groups were made).

Although no main effect of novelty was observed (*p* = 0.115), novelty interacted with age group, *F*(3, 413) = 3.20, *p* = 0.023, *ŋ*^2^ = 0.02 (see *Fig. *5). This interaction was followed up with three 2*2*2 ANOVAs investigating the effects of novelty whilst comparing older adults to the other age groups. For adolescents and older adults an interaction between novelty and age was observed, with adolescents remembering more words after exploring a novel versus familiar environment (i.e., a novelty bonus), and the reverse for older adults (a novelty detriment), *F*(1, 123) = 7.85, *p* = 0.006, *ŋ*^2^ = 0.06 (surviving Bonferroni-Holm correction α/3). For younger versus older adults a similar pattern was observed, with younger adults having higher word recall after exploration of a novel compared to familiar environment, and the reverse for older adults, *F*(1, 201) = 5.97, *p* = 0.015, *ŋ*^2^ = 0.03 (α/[3 − 1]). Finally, children also had higher word recall after exploration of a novel compared to familiar environment, while older adults showed the opposite pattern, *F*(1, 209) = 4.83, *p* = 0.029, *ŋ*^2^ = 0.02 (α/[3 − 2]). No other interactions were observed in the main ANOVA (all *p*s >  = 0.129).

As we did not check whether participants who completed the English version of the task were (near) native speakers, they may not have been sufficiently familiar with the words included in our list. We therefore repeated the main ANOVA without the data from the participants who completed the English version of the task and observed the same pattern of results as reported for the analyses on all subjects. Furthermore, when including language version as a between-subjects factor (Dutch; English), no effect of language was found.

### Word recognition

Figure 4B shows the corrected hit rate (CHR = hits – false alarms) on the recognition test per age group, novelty condition, and encoding type as a measure. Participants in the deep encoding condition recognized more words than participants in the shallow encoding condition, *F*(1, 424) = 43.74, *p* < 0.001, *ŋ*^2^ = 0.09. Age also influenced recognition, *F*(3, 424) = 16.85, *p* < 0.001, *ŋ*^2^ = 0.11. A quadratic relationship of age was observed, *Contrast estimate* = − 0.93, *p* < 0.001, with adolescents and younger adults remembering more words than older adults and children. Novelty did not influence CHR (*p* = 0.259), and no interactions were observed (all *p*s >  = 0.113).

### Exploration behavior

Figure 3A shows the map of one of the VEs and Fig. 3B a heatmap reflecting how often each area was visited including all participants. The heat map confirms that participants mostly stayed on the paths as instructed, although some deviations from the paths or shortcuts to other paths can be seen. After the second round of exploration, landmark memory was tested for the corresponding VE. The landmark memory analyses are reported in SI: Appendix 4, and analyses linking exploration behavior and landmark memory in SI: Appendix 5.

**Figure 6:**
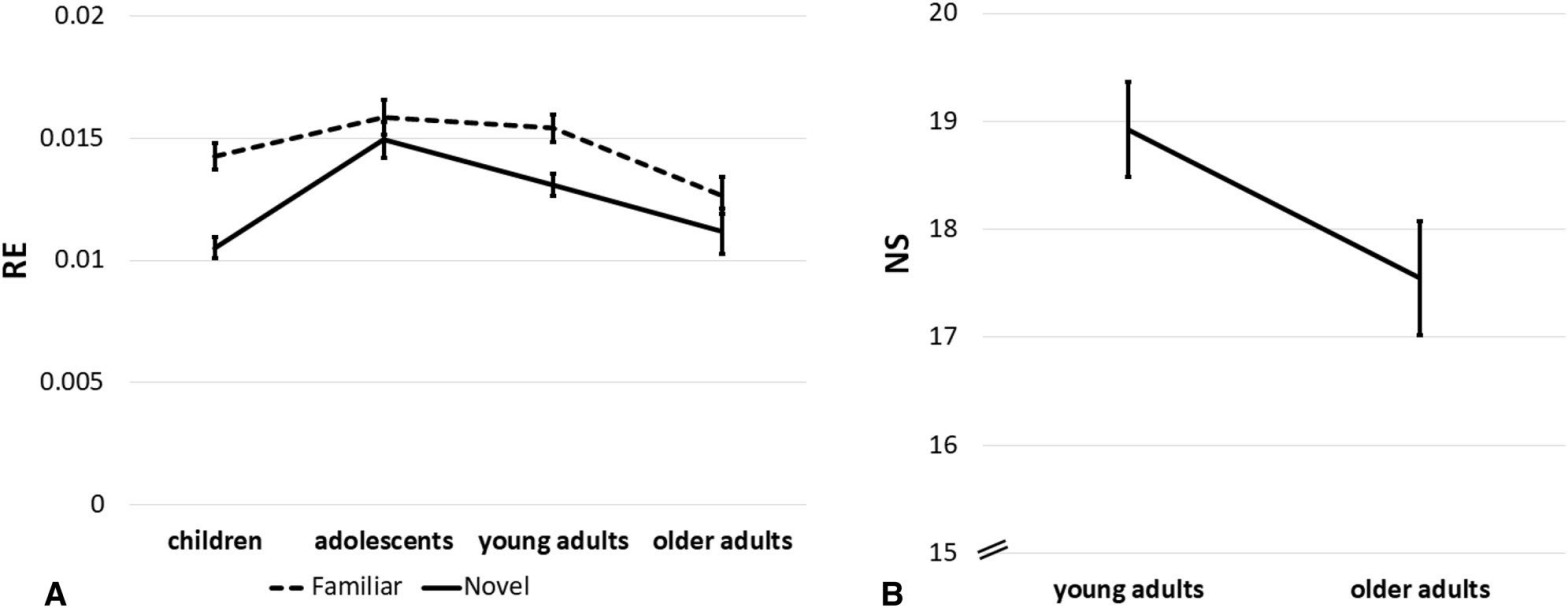
Roaming entropy (RE) and novelty seeking (NS) across age groups. Mean RE is shown for all age groups, including children, adolescents, younger adults, and older adults in the novel and familiar condition. Mean NS scores are shown for younger and older adults only (as no standardized measures were obtained for children and adolescents). Error bars reflect standard errors of the mean.

The different exploration measures (i.e., encountered landmarks, distance travelled, and RE in round 2) were analyzed with univariate ANOVAs with novelty (novel; familiar) and age (children; adolescents; younger adults; older adults) as factors. A main effect of age group was observed for the number of encountered landmarks, distance travelled, and RE in round 2, all *F*s > 6.06, *p*s < 0.001, *etas* > 0.04. Follow-up contrasts revealed quadratic effects for all three measures with higher exploration measures for adolescents and younger adults than for children and older adults (see Fig. 6 for RE across age groups), *Contrast estimates* < =|2.35|, *p*s <  = 0.002. No interactions between age group and novelty were observed for distance travelled or encountered landmarks (*p* = 0.656 and *p* = 0.499 respectively), but age and novelty interacted for RE in round 2, *F*(3, 428) = 2.73, *p* = 0.044, *ŋ*^2^ = 0.02. RE in round 2 was affected by novelty, with a larger RE in the familiar compared to the novel condition, *F*(1, 436) = 8.49, *p* = 0.004, *ŋ*^2^ = 0.02. For the number of encountered landmarks and distance travelled no effect of novelty was observed (*p*s > 0.060). There was a statistical trend suggesting that NS scores were higher for younger versus older adults, *t*(153.74) = 1.950, *p* = 0.053 (equal variances not assumed).

### RE, age, novelty, and encoding type predicting word recall

In line with the main ANOVAs a GLM (details in Methods) suggested that word recall was higher in the deep than in the shallow condition, and an effect of age group was found (see Table 1). Novelty did not show a significant effect. Extending the main analyses, we observed that RE in round 2 was predicting later memory success, with higher RE associated with better word recall.

**Table 1:** GLM predicting word recall.

	Word recall	b Value	t Value	*p* Value
	*df* = 380			
Novelty (novel-familiar)		0.16	1.66	.098
Encoding type (shallow-deep)		**−** **0.80**	**−** **7.98**	** < .001**
Age		**0.19**	**4.19**	** < .001**
RE round 2		**22.09**	**2.21**	**.028**

Encoding type and novelty acted as categorical predictors and RE for round 2 and age as continuous predictors in a GLM predicting word recall. The b values reflect the regressors’ slope (i.e., beta coefficients). Significant predictors are shown in bold.

## Discussion

In the current study we aimed to investigate whether beneficial effects of active exploration of novel environments on memory, that were previously observed in young adults, are also present in children and older adults. As previous work in rodents has suggested that novelty mainly promotes consolidation of weak memory traces, we set out to investigate whether depth of encoding is indeed a crucial factor in the effects of novelty on memory in humans too. In our task, participants either actively explored a novel or familiar VE before learning a list of words. After a short distractor task, we immediately tested word memory with a free recall and recognition test. Landmark memory was tested using old/new judgments and certainty estimates. Successful landmark memory was defined as the “sure” CHR.

In line with the rise and fall of the dopaminergic system, quadratic effects showed higher memory performance (word recall and recognition) in adolescents and young adults, compared to children and older adults^38,45,46^. Furthermore, we found that children, adolescents, and younger adults remembered more words after exploring a novel versus a familiar environment, while a novelty detriment was observed in older adults. The results in the younger groups are in line with our previous findings that exposure to spatial novelty can promote memory^20,22,37^, and extend previous findings by providing the first experimental evidence that the beneficial effects of novelty may disappear in older adults. We also observed that NS scores were lower for older versus younger adults. The present study’s results can be explained by the deterioration of the novelty-related pathways in the brain that are believed to drive the positive effects of novelty on memory in older versus younger individuals^5–9,33,34^. Note that our current cross-sectional (non-longitudinal) approach comparing age group does not allow us to infer whether the observed effects were driven by a detriment in performance in older adults or better performance in younger participants. However, crucially, the effects of age and novelty on recall cannot be explained by general arousal or mood reports of participants, as these affective measures did not differ between novelty conditions and did not follow the same pattern over the different age groups as the effects on memory. Irrespective of novelty, arousal and mood peaked in children, with higher ratings for children compared to adolescents. In line with previous work, we observed no novelty-related effects on recognition^10,20,37^. It is possible, however, that our subjective measure of arousal and mood (9-point SAMs) was not sensitive enough to identify fluctuations. We therefore recommend that future studies use more objective or validated measures, such as heart rate (variability), pupil size, or dedicated questionnaires (such as the Toronto Hospital Alertness Test;^56^) to estimate awareness.

A potential explanation for our findings is that novelty influenced motivation differently across age groups (as suggested by the NOvelty-related Motivation of Anticipation and exploration by Dopamine model [NOMAD]^32^. The mesolimbic dopamine system is strongly associated with motivation^57,58^, but this system also has been suggested to underlie the effects of novelty on memory^59,60^. As such, the effects of novelty through dopaminergic modulation as described above, would likely share neural pathways with potential effects of novelty on motivation. In the current study, however, we did not include a measure of motivation, nor did we include measures of dopaminergic functioning, therefore potentially differential effects of novelty on motivation and dopaminergic pathways cannot be teased apart.

One limitation of the current study was that the number of participants per age group varied, as we could not select participants on age in the museum setting (i.e., everyone of age 8 and above was allowed to participate). Due to natural variation in the age of the museum visitors, the older adult group was the smallest (*n* = 64), with some conditions having a low number of participants. Potentially, the lack of a novelty effect in this age group was due to this smaller sample size. Especially, identifying a 3-way interaction may have been hard as a result (observed power was moderate for the 3-way interaction = 0.458). It is also possible that participants in the different age groups experienced the environments differently in terms of novelty, potentially explaining the observed differences between the age groups. Future studies could include additional subjective measures regarding the experienced novelty of the environments to further address the effects of such differences over the lifespan.

One may argue that a limitation regarding our current interpretation in terms of dopaminergic mechanisms is that the mean age of our older adults was ~ 53 years old, while prior indications of age-related deterioration of dopaminergic pathways were based on slightly older participants (e.g., > 60 years;^61–63^. However, dopaminergic signaling has already been shown to go down in early adulthood^38^, and a reduction in cortical and striatal DA receptor density has been reported already in middle adulthood (~ 40 years;^64,65^, and is believed to decrease linearly over the adult lifespan^66,67^. Furthermore, structural changes in the substantia nigra and dopaminergic functioning have been shown to follow an inverted U-shape (including a rise during early development and a fall later in life) over the lifespan^68,69^, suggesting that changes in this region develop gradually. Taking into account this work on dopaminergic functioning across the lifespan, it is unlikely that dopamine deterioration occurs abruptly. Despite our sample of older individuals being relatively young, it is likely that some of these changes already started to develop in our sample too, as changes in dopaminergic functioning have already been reported in early adulthood. This point could be further investigated by including a sample of older individuals (e.g., > 60 and above) or by adopting a longitudinal design in future studies. Also note that our findings of novelty influencing immediate recall (only after a short distraction task) differ from the studies in animals, where typically long-term potentiation or memory are measured after longer delays (e.g., 24 h later). Due to this difference and other differences regarding the type of novelty (virtual environments versus real novel environments/stimuli) or memory content (word memory versus e.g., taste/scent recognition), it is difficult to compare the current findings with those in animals. As such it is not possible to make conjectures regarding underlying neural mechanisms on basis of the current study’s results. Future studies in humans could aim to further bridge the gap between the two literatures by using more similar designs as in the animal studies, or to investigate the potential neural mechanisms (such as identifying dopaminergic or noradrenergic modulations via pharmacological interventions).

We were uniquely able to link exploration behavior, as measured by RE to later memory success on an unrelated task. Participants who explored more showed higher subsequent word recall. These effects were found above and beyond the effects of novelty, which was also included in the model. It is possible that these differences in exploration behavior reflect individual differences, such that high explorers also generally have better memory, but it is also possible that exploration behavior had a positive effect on memory success by promoting motivation or arousal^15,18^. Note, however, that we did not observe effects of novelty on a subjective measure of arousal. Future studies could aim to manipulate exploration behavior experimentally to further address this point.

As expected, participants remembered and recognized more words in the deep compared to the shallow encoding condition. However, in contrast to expectations, no interaction between novelty and depth of encoding was found. A potential reason for this may be that we tested memory after a short distractor task, while effects of the level of encoding may rely on memory consolidation, which may take longer.

As could be expected, due to repeated potential exposure, landmark memory was better in the familiar than novel condition. Landmark memory peaked in adolescents. This may be explained by our finding that adolescents (and young adults) encountered more landmarks, had higher RE, and travelled further during the second round of exploration (novel/familiar). Indeed, encountered landmarks, distance travelled, and RE exhibited a positive correlation with later memory success on the landmark task. Interestingly, participants in the familiar condition had higher RE than those in the novel condition. A reason for this may be that we tested memory immediately after a short distractor task. Some animal studies suggest that the interaction of novelty and depth of encoding develops over time^46,70^, via dopaminergic neuromodulation of memory consolidation rather than dopaminergic “priming” of memory encoding^71^.

Taken together, our findings suggest that exploring a novel environment has a generalizable memory boosting effect, on both weakly and strongly encoded information, in children, adolescents, and younger adults, but not in older adults. These results imply that the beneficial effects of novelty on memory are limited to younger individuals, and that interventions aimed at counteracting age-related memory impairments may be less effective.

## Supplementary Information

Supplementary Information 1.

Supplementary Information 2.

Supplementary Information 3.

Supplementary Information 4.

Supplementary Information 5.

## Data Availability

Data and scripts used for the current article will be made available via the Open Science Framework (OSF) and additional information, including raw data files and tasks, will be shared via DataVerseNL and can be requested by contacting the corresponding author Dr. J. Schomaker.
