# Supplementary material for: Effects of exploring a novel environment on memory across the lifespan
Source: Sci Rep. 2022 Oct 5;12:16631. doi: 10.1038/s41598-022-20562-4 (PMC9533976; doi:10.1038/s41598-022-20562-4)
Supplement: Supplementary file 1 — Supplementary Information 1. [file 41598_2022_20562_MOESM1_ESM.docx]

**Supplementary information: Appendix 1**

| **Sex:** | **Female** | **Male** | **Other** |
| --- | --- | --- | --- |
|  | 208 | 233 | 0 |
| **Handedness:** | **Right** | **Left** | **Ambidextrous** |
|  | 388 | 44 | 9 |

*Table S1.1: Participant information*

| **Age group:** | **8-11** | **12-17** | **18-44** | **>44** |
| --- | --- | --- | --- | --- |
| *Deep* |  |  |  |  |
| Novel | 57 | 17 | 50 | 17 |
| Familiar | 54 | 26 | 52 | 25 |
| *Shallow* |  |  |  |  |
| Novel | 18 | 11 | 24 | 9 |
| Familiar | 24 | 13 | 29 | 13 |

*Table S1.2: Number of participants per age group and condition*


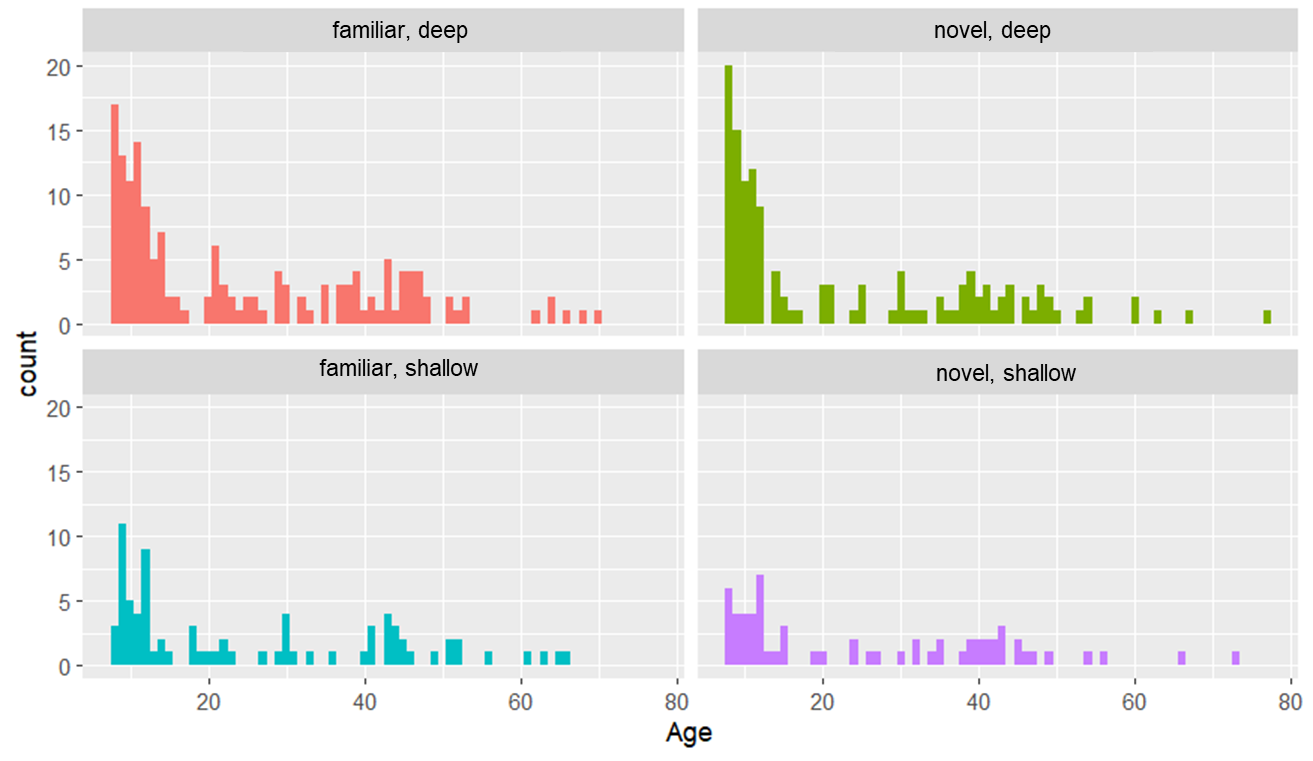


*Figure S4.* Number of participants per novelty and encoding type condition.
