# Supplementary material for: Effects of exploring a novel environment on memory across the lifespan
Source: Sci Rep. 2022 Oct 5;12:16631. doi: 10.1038/s41598-022-20562-4 (PMC9533976; doi:10.1038/s41598-022-20562-4)
Supplement: Supplementary file 2 — Supplementary Information 2. [file 41598_2022_20562_MOESM2_ESM.docx]

**Supplementary information: Appendix 2**

*Ratings*

The arousal and mood ratings were investigated with a mixed ANOVA with Time (pre; post) as a within-subjects factor and Novelty (novel; familiar), and Age group (children [age 8-11 years]; adolescents [age 12-17]; younger adults [age 18-44]; older adults [age >45]) as between-subjects factors. As we did not have a hypothesis regarding the effects on arousal or mood, a main effect of age was followed up by exploratory comparisons between children and adolescents, adolescents and younger adults, and younger and older adults. In addition, the effect of age was investigated with quadratic contrasts.

Figure S1 shows the arousal and mood ratings per age group. Arousal ratings did not change from the first to the second exploration phase (pre versus post; *p* = .438), and novelty did not influence the arousal ratings (*p* = .249). Arousal ratings differed among the age groups, *F*(3, 423) = 9.56, *p* < .001, *ŋ^2^* = .06. Post-hoc contrasts showed that children gave higher arousal ratings than adolescents, *F*(1, 220) = 6.27, *p* = .013, *ŋ^2^* = .03 (surviving Bonferroni-Holm correction at α/[3-2]), while arousal ratings were similar for adolescents and younger adults (*p*  = .428), and for younger and older adults (*p* = .249). No quadratic effect of age was found (*p* = .311). None of the factors interacted (all *p*s > .271).

Mood ratings did not differ after the first and second exploration phase (*p* = .107). Novelty also did not influence the mood ratings (*p* = .516 and *p* = .159 respectively), but age affected mood, *F*(2, 424) = 3.18, *p* = .042, *ŋ^2^* = .02. Post-hoc contrasts showed that children gave higher mood ratings than adolescents, *F*(1, 220) = 5.74, *p* = .017, *ŋ^2^* = .03, while mood ratings were similar for adolescents and younger adults (*p*  = .247), and younger and older adults (*p* = .703). This relationship between age and mood was also evidenced by a quadratic effect, *Contrast estimate* = 21.80, *p* = .011. None of the factors interacted (all *ps* > .211).


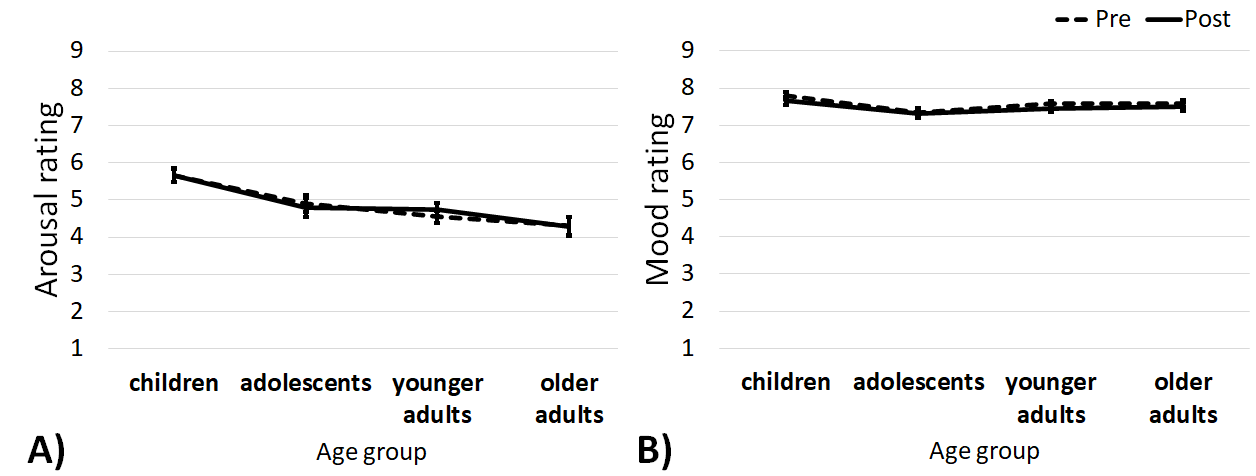


*Figure S2. Arousal and mood ratings.* A) Arousal and B) mood ratings on 9-point Likert scales for children (age 8-11), adolescents (age 12-17), younger adults (18-45) and older adults (46-77). Error bars reflect standard errors of the mean. Both arousal and mood ratings peaked in children, but neither novelty nor time (first or second exploration) influenced the ratings.
