# Supplementary material for: Effects of exploring a novel environment on memory across the lifespan
Source: Sci Rep. 2022 Oct 5;12:16631. doi: 10.1038/s41598-022-20562-4 (PMC9533976; doi:10.1038/s41598-022-20562-4)
Supplement: Supplementary file 3 — Supplementary Information 3. [file 41598_2022_20562_MOESM3_ESM.docx]

**Supplementary information: Appendix 3**

*Level-of-processing encoding task*

Performance on the word encoding task (response times [both correct and incorrect] and accuracy [hits]) were subjected to two 2*2*4 ANOVAs with Novelty (novel; familiar), Encoding type (shallow; deep) and Age group (children; adolescents; younger adults; older adults) as between-subject factors.

Participants in the deep encoding condition had higher accuracy on the encoding task than those in the shallow encoding condition, *F*(1, 394) = 10.21, *p* < .001, *ŋ^2^* = .03. Novelty did not influence accuracy on the encoding task (*p* = .251). Accuracy, however, differed as a function of age group, *F*(3, 394) = 4.26 *p* = .006, *ŋ^2^* = .03. A quadratic relationship of age was observed, *Contrast estimate* = -0.34, *p* = .011, with the adolescents and younger adults having higher accuracy than the children and older adults.

Learning type also interacted with age group, *F*(3, 394) = 3.55, *p* = .015, *ŋ^2^* = .03. This interaction was followed up with 2*2*2 ANOVAs investigating the effects of learning type whilst comparing older adults to the other age groups. For younger and older adults a statistical trend effect was observed for an interaction between learning type and age was observed, with older adults in the deep encoding having higher accuracy than those in the shallow encoding condition, and the reverse for younger adults, *F*(1, 190) = 5.78, *p* = .017, *ŋ^2^* = .03, but this did not survive Bonferroni-Holm correction (α/3). There was no interaction between age group and learning type for children and older adults (*p* = .576) nor for adolescents and older adults (*p* = .185). In the main ANOVA no other interactions were observed (all *p*s >= .251).

Learning type did not influence response times during the encoding (*p* = .294), nor did novelty (*p* = .496). There was an effect of age group, *F*(3, 394) = 12.31, *p* < .001, *ŋ^2^* = .09, with older individuals being slower than younger, but no evidence of a quadratic relation (*p* = .937). No interactions were found (*p*s >= .277).
