# Supplementary material for: Effects of exploring a novel environment on memory across the lifespan
Source: Sci Rep. 2022 Oct 5;12:16631. doi: 10.1038/s41598-022-20562-4 (PMC9533976; doi:10.1038/s41598-022-20562-4)
Supplement: Supplementary file 4 — Supplementary Information 4. [file 41598_2022_20562_MOESM4_ESM.docx]

**Supplementary information: Appendix 4**

*Landmark memory*

The ‘sure’ CHR for the landmarks was investigated with a 2*4 ANOVA with Novelty (novel; familiar) and Age Group (children; adolescents; younger adults; older adults) as factors. Again, a main effect of age was further investigated with a quadratic contrast.

Figure 5 shows an estimate of recollection (“sure” CHR) for the landmarks per age group, novelty condition, and encoding type. As could be expected due to potential repeated exposure, memory for the landmarks as measured by the ‘sure’ CHR was higher in the familiar compared to the novel condition, *F*(1, 323) = 51.35, *p* < .001, *ŋ^2^* = .14. Age influenced the ‘sure’ CHR as well, *F*(3, 323) = 4.91, *p* = .002, *ŋ^2^* = .04. The ‘sure’ CHR was highest for adolescents, and lower for children, younger adults, and older adults, as evidenced by a quadratic relationship, *Contrast estimate* = -0.94, *p* < .001. There was no significant interaction between age group and novelty (*p* = .768).


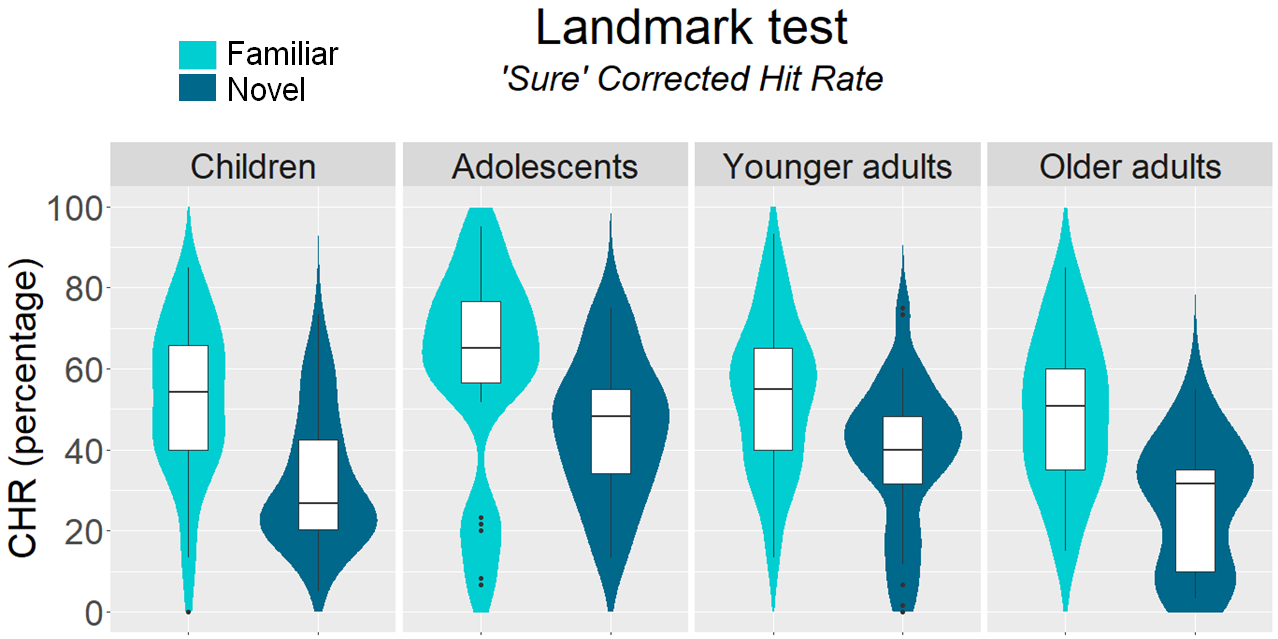


*Figure S4. Memory performance on landmark task.* ‘Sure’ corrected hit rate (CHR) in percentages as an estimate of landmark recollection for children (age 8-11), adolescents (age 12-17), younger adults (age 18-45) and older adults (age 46-77) after exploring a novel or familiar environment.
