# Supplementary material for: Effects of exploring a novel environment on memory across the lifespan
Source: Sci Rep. 2022 Oct 5;12:16631. doi: 10.1038/s41598-022-20562-4 (PMC9533976; doi:10.1038/s41598-022-20562-4)
Supplement: Supplementary file 5 — Supplementary Information 5. [file 41598_2022_20562_MOESM5_ESM.docx]

**Supplementary information: Appendix 5**

*Exploration behavior and landmark memory*

To investigate the link between exploration behavior as measured by the number of encountered landmarks in the second exploration round (the round for which memory was tested) and subsequent landmark memory success, a correlation between the number of encountered landmarks and the “sure” CHR was calculated.

A positive relationship between RE (a measure of exploratory behavior) for exploration round 2 and “sure” CHR for landmarks was observed, *r*(425) = .632, *p* < .001, which makes sense as a higher RE reflects more exploration and thus higher chances of encountering landmarks that could be remembered. A similar positive correlation was found between the number of encountered landmarks during round 2 and later memory success on the landmark task as quantified by the “sure” CHR, *r*(306) = .480, *p* < .001. Distance travelled in round 2 also had a positive correlation with “sure” CHR measure of landmark memory, *r*(273) = .428, *p* < .001. Figure 7 shows the positive relationships between the “sure” CHR for landmarks and A) RE, B) encountered landmarks, and C) distance travelled during exploration round 2.


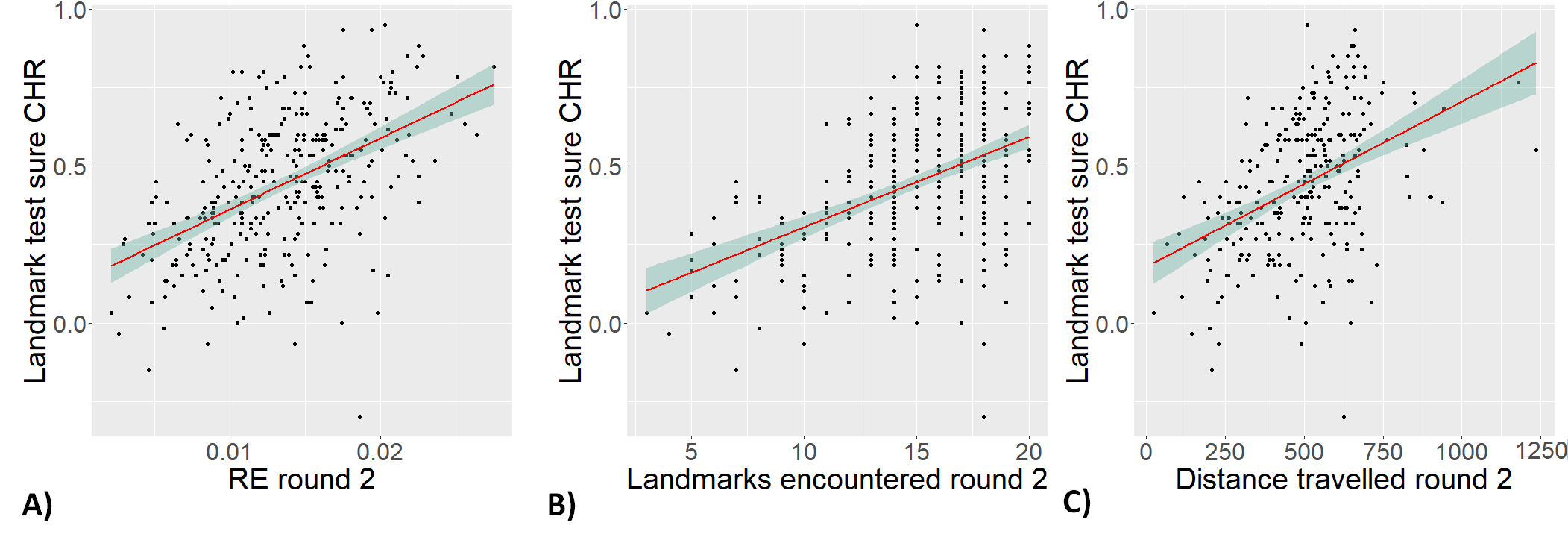


*Figure S5. Landmark memory and exploration.* The relationship between later memory success on the landmark task as quantified by the “sure” CHR on the landmark task and A) roaming entropy (RE), B) the number of encountered landmarks, and C) the distance travelled during exploration round 2. Note that the “sure” CHR for the landmark task could be negative for participants with more false alarms than “sure” hits. In all panels a confidence interval is shown as the shaded area around the linear trendline.
